# *Novel C12orf65* mutations in patients with axonal neuropathy and optic atrophy

**DOI:** 10.1136/jnnp-2013-306387

**Published:** 2013-11-06

**Authors:** Arianna Tucci, Yo-Tsen Liu, Elisabeth Preza, Robert D S Pitceathly, Annapurna Chalasani, Vincent Plagnol, John M Land, Daniah Trabzuni, Mina Ryten, on behalf of UKBEC, Zane Jaunmuktane, Mary M Reilly, Sebastian Brandner, Iain Hargreaves, John Hardy, Andrew B Singleton, Andrey Y Abramov, Henry Houlden

**Affiliations:** 1 Department of Molecular Neuroscience and Reta Lila Weston Research Laboratories, UCL Institute of Neurology and The National Hospital for Neurology and Neurosurgery, London, UK; 2 MRC Centre for Neuromuscular Diseases, UCL Institute of Neurology and The National Hospital for Neurology and Neurosurgery, London, UK; 3 Section of Epilepsy, Department of Neurology, Neurological Institute, Taipei Veterans General Hospital, Taipei, Taiwan; 4 National Yang-Ming University School of Medicine, Taipei, Taiwan; 5 Neurometabolic unit, UCL Institute of Neurology and The National Hospital for Neurology and Neurosurgery, London, England; 6 UCL Genetics Institute (UGI), London, UK; 7 Department of Genetics, King Faisal Specialist Hospital and Research Centre, Riyadh, Saudi Arabia; 8 Division of Neuropathology, The National Hospital for Neurology and Neurosurgery, London, UK; 9 Laboratory of Neurogenetics, National Institute of Aging, National Institutes of Health, Bethesda, Maryland, USA

**Keywords:** Genetics, Neuropathy

## Abstract

**Objective:**

Charcot-Marie Tooth disease (CMT) forms a clinically and genetically heterogeneous group of disorders. Although a number of disease genes have been identified for CMT, the gene discovery for some complex form of CMT has lagged behind. The association of neuropathy and optic atrophy (also known as CMT type 6) has been described with autosomaldominant, recessive and X-linked modes of inheritance. Mutations in Mitofusin 2 have been found to cause dominant forms of CMT6. Phosphoribosylpyrophosphate synthetase-I mutations cause X-linked CMT6, but until now, mutations in the recessive forms of disease have never been identified.

**Methods:**

We here describe a family with three affected individuals who inherited in an autosomal recessive fashion a childhood onset neuropathy and optic atrophy. Using homozygosity mapping in the family and exome sequencing in two affected individuals we identified a novel protein-truncating mutation in the C12orf65 gene, which encodes for a protein involved in mitochondrial translation. Using a variety of methods we investigated the possibility of mitochondrial impairment in the patients cell lines.

**Results:**

We described a large consanguineous family with neuropathy and optic atrophy carrying a loss of function mutation in the *C12orf65* gene. We report mitochondrial impairment in the patients cell lines, followed by multiple lines of evidence which include decrease of complex V activity and stability (blue native gel assay), decrease in mitochondrial respiration rate and reduction of mitochondrial membrane potential.

**Conclusions:**

This work describes a mutation in the C12orf65 gene that causes recessive form of CMT6 and confirms the role of mitochondrial dysfunction in this complex axonal neuropathy.

## Introduction

Inherited neuropathies are the most common genetic neurological disorders and affect ∼1 in 2500 individuals.^1^ Charcot–Marie Tooth (CMT) includes a group of hereditary disorders in which motor and sensory neuropathy is the sole or primary part of the disease. CMT is traditionally classified into two types based on electrophysiological and neuropathological criteria, where CMT1 defined as ‘demyelinating’ and CMT2 as ‘axonal’.^2^ CMT can be associated with a variety of additional clinical features. The association between hereditary motor and sensory neuropathy and optic atrophy, also known as CMT6 (OMIM #601152), has been reported in families with different modes of inheritance, comprising over 50 cases.^3–8^ The clinical manifestations of CMT6 consist of distal muscle weakness and wasting starting in the lower limb with reduced reflexes and glove and stocking sensory loss. There is progressive visual acuity loss due to optic atrophy, eventually leading to blindness. The age at onset is usually in childhood for the neuropathy and in the second decade for the optic atrophy.

Autosomal dominant CMT6 has previously been found to harbour mutations in the Mitofusin 2 (*MFN2*) gene.^9^ X-linked recessive mutations have been identified in the phosphoribosylpyrophosphate synthetase I (*PRPS1*) gene in CMT6 families.^10^ Conversely, no genetic cause for the autosomal-recessive forms of CMT6 has been identified until the work described here, where we identify a novel mutation in the *C12orf65* gene and then characterise this mitochondrial protein in CMT6.

## Methods

### Samples

DNA samples from multiple individuals in a large Indian family with CMT6 were collected at the Royal Free Hospital, London. An additional 93 probands affected by complex axonal neuropathy (axonal neuropathy plus one of the following: optic atrophy, retinitis pigmentosa, psychomotor retardation, cerebellar ataxia or pyramidal signs) were collected at the National Hospital for Neurology and Neurosurgery (NHNN), London. All patients were negative for known mutations in the following genes *MFN2*, *MPZ*, *GJ1B, BSCL2* and *NEFL.* Additional 90 samples with axonal neuropathy and evidence of mitochondrial impairment on muscle biopsy were collected from the neurometabolic unit at the NHNN. Genomic DNA was purified from peripheral blood cells using standard procedures.

### Nerve biopsy

The nerve biopsy soon after the surgical removal was immersed in a fixative for overnight containing 3% buffered glutaraldehyde and 0.2M sodium cacodylate buffer. Then, specimens were cut with razor in 1–2 mm thick pieces and osmicated in secondary fixative—osmium tetroxide. After fixation, the specimens were impregnated into epoxy resin from which semithin (∼1 µ) sections or ultrathin (∼70 nm) sections were cut and put on glass slides or grids, respectively. Semithin sections were stained either with Methylene blue-Azure A and basic fuchsin or Toluidine blue and examined by light microscopy on various magnifications. Ultrathin (∼70 nm) sections were stained with uranyl acetate/lead citrate and examined by electron microscopy on various magnifications.

### Single Nucleotide Polymorphism genotyping and autozygosity mapping

Genome-wide SNP genotyping was performed at National Institute of Health (NIH) (Bethesda, USA). Each individual was assayed on a Illumina HumanHap300 BeadChip, yielding approximately 317 000 SNPs. Samples were processed, hybridised and scanned following the instructions of the manufacturer. Clustering, normalisation and genotype calls were performed using the GenomeStudio 2010.3 Genotyping Module (Illumina). Regions of shared homozygosity that segregated with disease were visually identified using the Illumina Genome Viewer tool within the BeadStudio suite.

Whole-exome sequencing was carried out at NIH (Bethesda, USA). Nimblegen SeqCap EZ Exome (in solution capture) Kit was used for the exome capture. Sequencing was performed on a Genome Analyzer IIx, according to the manufacturer’s instruction. Raw sequencing reads were aligned to the hg18 build of the reference genome using the software Novoalign. Calling was performed using Samtools V.0.18 and the resulting calls were annotated using ANNOVAR.

### Mutation validation and screening in the additional cohorts

Primers for PCR amplification were designed by Generunner (http://www.generunner.net/). After PCR amplification, coding regions and exon–intron junctions of *C12orf65* gene were sequenced by Sanger's sequencing, using the Big Dye Terminator Cycle Sequencing Kit V3 (Applied Biosystems, Foster City, California, USA) and analysed by a 3130 Genetic Analyser sequencing machine (Applied Biosystems). Sequences were examined in silico for mutations by Sequencher software 4.9 (Gene Codes Corporation, Ann Arbor, Michigan, USA).

### Lymphoblast cell cultures

Lymphoblast cells were obtained from Case 1 and two unaffected relative carriers. Lymphoblastoid cell lines were established by Epstein–Barr virus transformation of lymphocytes isolated from peripheral blood. Cell lines were stored at the European Collection of Cell Cultures. Informed consent was obtained for all samples. Patient and control lymphoblasts were thawed and maintained in culture in modified RPMI-1640 medium containing 300 mg/L L-Glutamine and HEPES (Invitrogen) supplemented with 10% heat-inactivated Fetal Bovine Serum (FBS) (Invitrogen) at 37°C and 5% CO_2_. Fresh medium was added every 3 days and cultures were expanded accordingly.

### Transcript analyses

Purification of total RNA was performed using the Qiagen miRNeasy Mini Kit, Hilden, Germany (catalogue #217004). cDNA synthesis was performed using Qiagen Omniscript RT Kit, Hilden, Germany (catalogue #205110). Multiplex quantitative real-time PCR assays for the levels of C12orf65 and RPL13A were performed using SYBR Green PCR Master Mix Kit (Applied Biosystems) with a Corbett Rotor-Gene real-time quantitative thermal cycler (Corbett Research/Qiagen). Thermal cycling and gene-specific primers are available upon request. Relative quantity of each C12orf65 level was normalised by RPL13A.

### Expression profiling of C12orf65in human Central Nervous System tissue

Expression data were generated from control human central nervous system (CNS) tissue using Affymetrix Exon 1.0 STArrays. CNS samples were collected by the Medical Research Council Sudden Death Brain and Tissue Bank, Edinburgh, UK,^11^ and the Sun Health Research Institute, an affiliate of Sun Health Corporation, USA.^12^ A full description of the samples used and the methods of RNA isolation and processing can be found in Trabzuni *et al*,^13^ and Trabzuni *et al*.^14^ All arrays were preprocessed with robust multiarray average quantile normalisation with GC background correction^15^ and log2 transformation in Partek's Genomics Suite V.6.6 (Partek, St. Louis, Missouri, USA). Regional differences in gene-level expression were investigated with Partek's mixed-model ANOVA is the name for a statistical test, and gender and batch effects (date of hybridisation and brain bank) were included as cofactors.

### Blue native in-gel complex V assay

The mitochondrial fraction was obtained from the lymphoblastoid cell pellets (7.5×10^5^ cells) using two low-speed centrifugation steps (600 g×10 min) at 4°C, separated by a homogenisation step. Then, the mitochondrial membranes were solubilised with a 750 mM amino hexanoic acid/50 mM Bis Tris buffer + 4% n-dodecyl β-D maltoside detergent. Samples were left on ice for 30 min and a further high spin (14 000 g×10 min) was used to pellet insoluble material. An equal quantity of mitochondrial protein was loaded from each sample. Blue Native (BN) gel was run as previously described,^16^ using a 3–12% Bis–Tris gel (Invitrogen) to ensure discrete separation of complex V. Complex V activity was measured in a reverse direction. Complex V assay was performed by incubating the gel overnight in stain containing 34 mM Tris, 270 mM glycine, 14 mM magnesium chloride, 6 mM lead (II) nitrate and 8 mM ATP.

### O_2_ consumption

To measure mitochondrial respiration rate in intact cells, approximately 1×10^7^ cells were suspended in respiration medium (HBSS with 10 mM D-glucose) in a Clark-type oxygen electrode thermostatically maintained at 37°C. The oxygen electrode was calibrated with air-saturated water, assuming 406 nmol O_2_ atoms/mL at 37°C. Oxygen consumption was measured over 10 min with addition of oligomycin (final concentration 2 μg/mL) and FCCP (0.5 μM). All data were obtained using an Oxygraph Plus System (Hansatech Instruments, UK) with chart recording software.

For measurements of mitochondrial membrane potential (Δψ_m_), cells were loaded with 25 nM tetramethylrhodamine methylester (TMRM) for 30 min at room temperature in HBSS (156 mM NaCl, 3 mM KCl, 2 mM MgSO_4_, 1.25 mM KH_2_PO_4_, 2 mM CaCl_2_, 10 mM glucose and 10 mM HEPES, pH adjusted to 7.35), and the dye was present during the experiment. TMRM is used in the redistribution mode and therefore a reduction in TMRM fluorescence represents Δψ_m_ depolarisation. Z-stack images were obtained for accurate analysis. The values for wild-type (WT) were set to 100% and the other genotypes were expressed relative to WT^17^

Confocal images were obtained using a Zeiss 710 LSM with a 40× oil immersion objective. TMRM was excited using the 560 nm laser and fluorescence was measured at 580 nm.

## Results

### Clinical details

**Figure 1:**
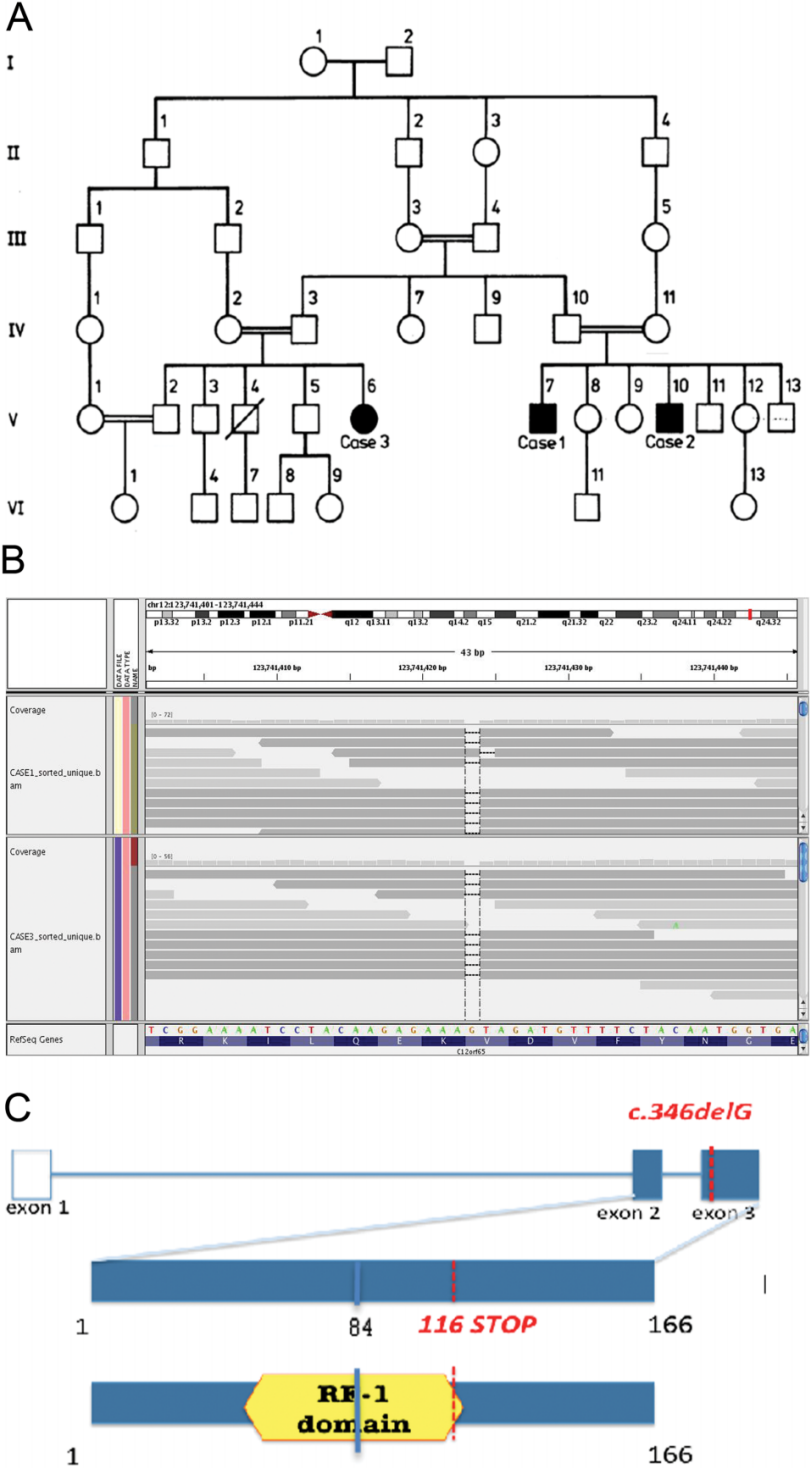
Pedigree of the family and the *C12orf65* p.V116X mutation. (A) Pedigree of the family. A square represents a male person and a circle represents a female person. A double horizontal line indicates parental consanguinity. Black symbols indicate affected members with Charcot–Marie Tooth type 2 (CMT2) and optic atrophy in whom a neurological exam was performed; blank symbols show unaffected family members. A diagonal line marks deceased individuals. This pedigree has been reported elsewhere.^25^ (B) Upper panel: whole-exome sequencing data for the p.V116X mutation in *C12orf65*. The aligned reads viewed through the Integrative Genomics Viewer (IGV) viewer (http://www.broadinstitute.org/igv/). Reads are depicted as arrows (grey bands). A coverage histogram per base is shown above the reads. RefSeq gene (over the *C12orf65* gene) is represented in the lower part both as amino acid sequence (blue) and as reference sequence: green, A; orange, G; red, T; blue, C. Dashes represent the deleted base. Note the drop of coverage (black arrow) over the dashed base, representing the G base deleted in both samples. (C) Schematic diagram of the *C12orf65* gene and protein. The position of the mutation in the patient DNA and the position of the resulting premature stop codon are indicated in red; blue indicates the mutation and the position of the resulting stop codon described in patients with severe encephalomyopathy.

Three members of a large consanguineous Indian family (figure 1A), affected by axonal neuropathy (table 1) and optic atrophy, were identified at the London Royal Free Hospital. Case 3 was first seen at the age of 35 years and then again at the age of 51 years. She is the first cousin of Cases 1 and 2 and presented as a child with delayed milestones, later at the age of 9 years she had lower limb and at 11 years upper limb weakness, along with static cognitive problems and visual difficulties. The examinations at the age of 35 and 51 years were remarkably similar with only mild deterioration of her clinical features. She had no dysmorphic features, but severe bilateral optic atrophy and static but significant cognitive problems. She had a brisk jaw jerk and a pout reflex. Marked distal symmetrical weakness and wasting affecting the limbs. In contrast to her cousins, tone in her upper and lower limbs was reduced with severe distal weakness in upper and lower limbs. Upper limb reflexes were normal. Knee and adductor reflexes were abnormally brisk. Ankle and plantar reflexes were absent. She had a moderately severe thoracic scoliosis. Sensation was impossible to assess reliably due to cognition but was likely to be abnormal. As a crude estimate of visual acuity, they were able to watch television and recognise faces across the room and she could do needlework.

**Table 1:** Nerve conduction studies

	Case 1	Case 2	Case 3	V-1	IV-II
SAP amplitude (uV):					
Median	10	NA	15	40	NA
Ulnar	6	NA	12	20	16
Sural	2	NA	5	NA	NA
MCV (m/s):					
Ulnar	41	46	51	54	60

NA, not available; SAP, sensory action potentials, median and ulnar SAPs were recorded from second and fifth fingers to wrist; MCV, motor conduction velocity, recorded over abductor digiti minimi (Case 1, V-1 and IV-II) and flexor carpi ulnaris (Cases 2 and 3).

Case 1 noted distal wasting and weakness at the age of 8, which slowly spread proximally. When he was examined (at the age of 34), he had severe muscle wasting of lower and upper limbs, bilateral optic atrophy and macular colloid bodies. Pyramidal signs were present in the upper limbs. Case 3 presented with a similar syndrome characterised by a very slowly progressing axonal neuropathy (onset in childhood), bilateral optic atrophy and pyramidal signs. This CMT6 family was originally reported as a case report in 1987 when the family members were in their middle 30s.^14^

### Nerve biopsy

**Figure 2:**
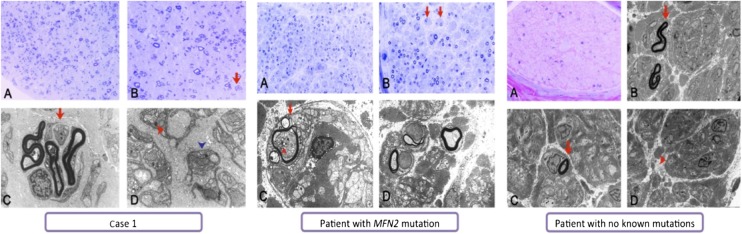
Sural nerve biopsies in affected case. Sural nerve biopsies (light and electron microscopy). Case 1. Semithin resin section stained with toluidine blue (A and B) shows markedly reduced numbers of large myelinated fibres accompanied by numerous small myelinated fibres mainly associated with regeneration clusters (arrow in B). Ultrastructural assessment (C and D) further highlights frequent regeneration clusters (arrow in C). The evidence of decreased numbers of unmyelinated fibres is confirmed by increased amounts of endoneural collagen and the formation of collagen pockets (red arrowhead in D) among flattened Schwann cell profiles (blue arrowhead in D). Scale bar: 60 μm (A), 30 μm (B) and 2 μm (C and D). Patient with *MFN2* mutation. Semithin resin section stained with toluidine blue (A and B) shows markedly reduced numbers of large myelinated fibres accompanied by occasional regeneration clusters and occasional fibres surrounded by concentric Schwann cell profiles forming onion bulb-like structures (arrows in B). Ultrastructural assessment (C and D) confirms that myelinated fibres are markedly reduced in numbers and shows no evidence of active or chronic demyelination; instead it reveals pseudo-onion bulbs indicative of regeneration (arrow in C). The depicted mitochondria on the transverse sections of the nerve fascicles show no obvious ultrastructural abnormality, although some intra-axonal clusters of mitochondria are occasionally observed (arrowhead in C). Scale bar: 80 μm (A), 40 μm (B) and 5 μm (C and D). Patient with no known mutation. Semithin resin section stained with methylene blue azure-basic fuchsin (MBA-BF) (A) shows markedly reduced numbers of large myelinated fibres with no apparent evidence of regeneration and no evidence of active macrophage-associated demyelination or chronic demyelinating/remyelinating process. Electron microscopy (B, C and D) confirms the markedly reduced numbers of large and small myelinated fibres (arrows in B and C). The unmyelinated fibres in contrast are better preserved (arrowhead in D). Scale bar: 40 μm (A) and 5 μm (B, C and D).

Light microscopy analysis of sural nerve biopsy from Case 1 showed a marked reduction in large myelinated fibres. The fascicles were populated by numerous small myelinated fibres, the majority of which were associated with regeneration clusters. Ultrastructural assessment confirmed the absence of both active and chronic demyelination and showed no evidence of ongoing axonal degeneration. The remaining myelinated fibres revealed normal thickness and morphology of the myelin. Loss of unmyelinated fibres was indirectly confirmed by increased amounts of endoneural collagen with the formation of collagen pockets among flattened Schwann cell profiles (figure 2).

### Genetic analyses

**Figure 3:**
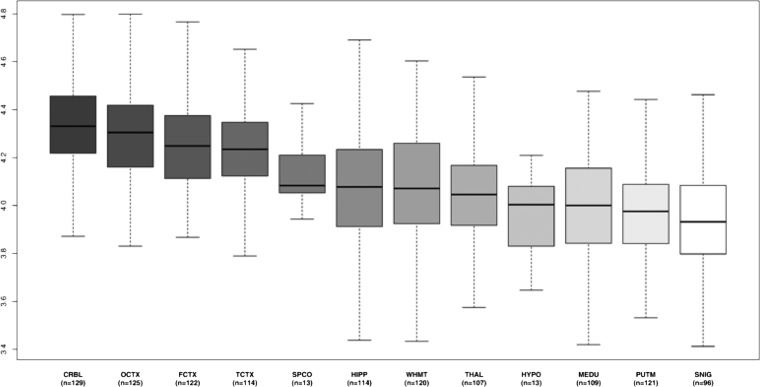
Expression of C12orf65 in 12 central nervous system (CNS) regions. (A) Box plot of C12orf65 mRNA expression levels in 12 CNS regions. The expression levels are based on exon array experiments and are plotted on a log2 scale (y axis). This plot shows significant variation in C12orf65 transcript expression across the 12 CNS regions analysed: putamen (PUTM, n=121), frontal cortex (FCTX, n=122), temporal cortex (TCTX, n=114), hippocampus (HIPP, n=114), cervical spinal cord (SPCO, n=13), substantia nigra (SNIG, n=96), hypothalamus (HYPO, n=13), medulla (specifically inferior olivary nucleus, MEDU, n=109), intralobular white matter (WHMT, n=120), thalamus (THAL, n=107) and cerebellar cortex (CRBL, n=129). Whiskers extend from the box to 1.5× the IQR.

Autozygosity mapping on two affected cousins (Case 1 and Case 3) identified five homozygous chromosomal segments (>1 Mb), concordant in both cousins encompassing 15.8 Mb and containing 226 genes (NCBI build 37.2). Whole-exome sequencing was used to perform a comprehensive search for pathogenic mutations in both cousins. The target region was sequenced at an average sequence depth of 26.6 for all samples. To identify potential causal variants, we selected all coding variants in the homozygous regions present in both samples. Then, we filtered them by discarding (i) variants documented in the dbSNP and the 1000-Genomes Project and (ii) synonymous substitutions. Five variants passed these filters: two were missense, one was a non-frameshift deletion and one was a nonsense mutation. The latter is a 1 bp deletion in *C12orf65* gene, resulting in a premature stop codon (NM_001143905: c.346delG: p.V116X) (figure 1B). As mutations in *C12orf65* have been recently described to cause a severe encephalomyopathy^17^ as well as spastic paraplegia and optic atrophy,^18^ we assessed segregation of this mutation in the family. The deletion was homozygous in the affected cases, and either absent or heterozygous in the unaffected relatives (figure 3). To further investigate the presence of the mutation in patients with complex neuropathy, we sequenced the coding exons of *C12orf65* in an additional cohort of 183 patients. None of the patients harboured potentially pathogenic variants.

To determine whether the truncating mutation induced mRNA nonsense-mediated decay, we tested the level of expression of C12orf65. This analysis showed that level of the C12orf65 mRNA was not reduced in the patients’ cells versus controls (data not shown).

**Figure 4:**
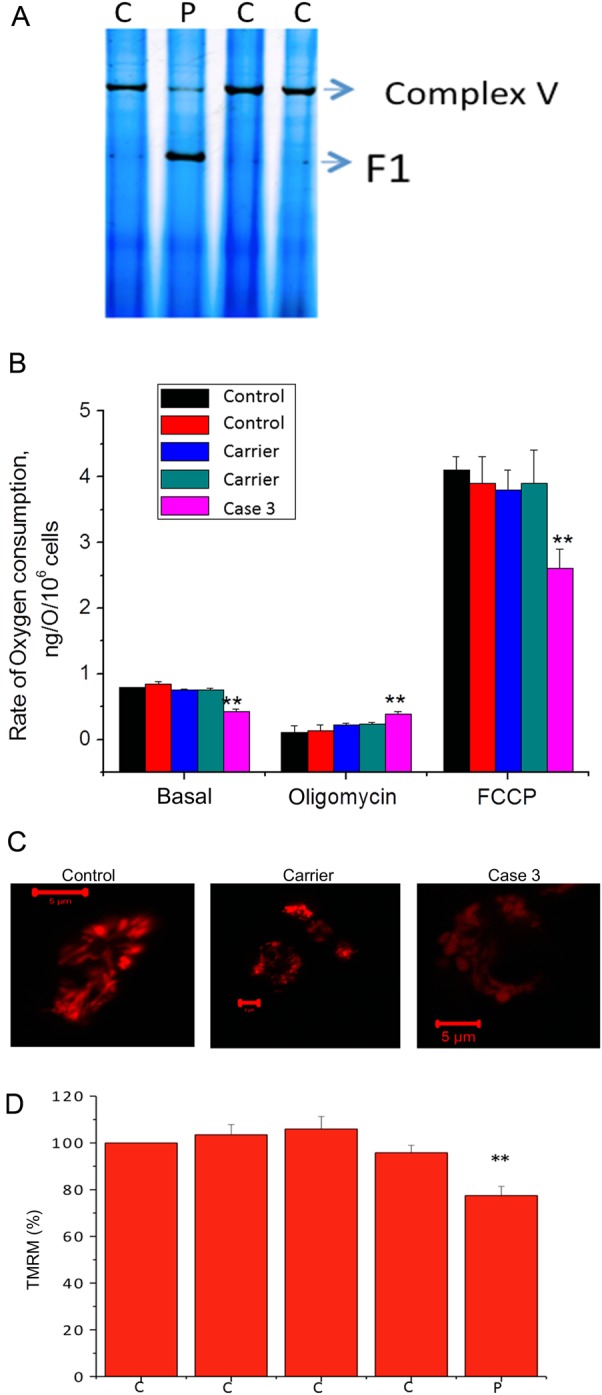
Mitochondria impairment in the patient's lymphoblasts. (A) BN-PAGE (in gel activity) shows reduced complex V activity in Case 3 compared with controls in lymphoblasts (demonstrated by reduced band density). V (F1-Fo)=complex V holeoenzyme; F1=catalytic site of complex V only. (B) Oxygen consumption in Case 3 and controls. Oxygen consumption was measured in immortalised lymphocytes using a Clark oxygen electrode. Respiration was inhibited by blocking ATP production using oligomycin (2 μg/mL) and maximised by adding the uncoupler FCCP (1 μM). Data are represented as mean±SEM. (C) and (D) Mitochondrial membrane potential. Mitochondrial membrane potential in control, carrier and Case 3 lymphoblasts was determined by tetramethylrhodamine methylester (TMRM) fluorescence. Control was taken as 100%. On the x-axes, C = controls; P = patient. **indicates p<0.01 compared to wild-type values.

*C12orf65* is a nuclear gene that encodes a mitochondrial matrix protein that appears to contribute to mitochondrial translation. Nonsense mutations in this have been found in patients with a mitochondrial disease associated with combined oxidative phosphorylation enzyme (OXPHOS) deficiency.^17^ Patients’ cells were analysed by BN-PAGE analysis to study mitochondrial respiratory chain protein complexes. This analysis showed a decreased activity of complex V as well as a defect in assembly and stability of complex V in the patient sample compared with controls (figure 4A).

In order to investigate the effect of mutations on mitochondrial respiration, we measured the rate of oxygen consumption in lymphocytes. The basal oxygen consumption in Case 3 cells was significantly reduced compared with control cells. Oligomycin (inhibitor of complex V) inhibited the respiration in control lymphocytes but to a significantly lesser extent in patients’ cells (p<0.001; figure 4B), confirming the decreased activity of complex V in Case 3 cells. Addition of 1 μM of the uncoupler FCCP accelerated respiration to maximal levels in control lymphocytes but to a significantly lesser degree in Case 3 cells, suggesting that the activity of the respiratory chain in these cells is limited.

Mitochondrial membrane potential (Δψm) is a major indicator of mitochondrial function and health. Δψm in Case 3 and control lymphoblasts was assessed by the fluorescent indicator tetramethylrhodamine methyl ester, TMRM. Case 3 cells were associated with a significant reduction in the TMRM signal (and hence in Δψ_m_) compared with controls. There were no differences between cell groups in mitochondrial morphology (figure 4C).

## Discussion

The present study describes a novel homozygous mutation in the *C12orf65* gene in patients with neuropathy and optic atrophy consistent with slowly progressive CMT6. The clinical phenotype is similar to the other CMT6 family members reported, but there are additional cognitive features and the disease is more slowly progressive with family members living a likely normal lifespan. Mutations in *C12orf65* have been previously described in children with Leigh syndrome, optic atrophy and ophthalmoplegia,^17^ as well as in patients with SPG55,^18^ also characterised by neuropathy and optic atrophy, but as opposed to the cases presented here they had no cognitive impairment and a less severe phenotype.

These data suggest a genotype–phenotype correlation, where the mutation site correlates with disease severity. Indeed *C12orf65* encodes for a 166 amino acid protein involved in protein translation termination and contains one RF-1 domain, found also in peptide chain release factors (figure 1C). The patients with a severe infanthood encephalomyopathy and death as a child carry mutations in *C12orf65* interrupting the RF-1 domain (p.L84X).^17^ Conversely, mutations sparing the RF-1 domain seem to cause a milder phenotype as reported in the cases presented here as well as in the SPG55 cases. Of note, the mutation site in our cases (p.V116X) would suggest a shorter protein as opposed to the SPG55 cases (p.R132X) and therefore a more severe phenotype.

We also investigated mitochondrial function in patient cell lines and showed that this was impaired at multiple levels of mitochondrial function. Mitochondrial membrane potential as well as mitochondrial respiration rate were reduced; BN gel analysis showed a decrease in complex V activity as well as a defect in the assembly of the complex.

These results highlight the importance of mitochondrial dysfunction in peripheral nerve disease and in optic atrophy. In peripheral nerve disease, the mitochondrial dysfunction is frequently associated with axonal CMT2. Indeed Mitofusin 2, the major cause of dominant CMT2, is a mitochondrial membrane protein involved in mitochondrial fusion^19^ and in the regulation of mitochondrial membrane potential and the OXPHOS system.^20^ Furthermore mutations in GDAP1, which encodes a mitochondrial membrane protein, and mutations in MT-ATP6, which encodes the ATP6 subunit of the mitochondrial ATP synthase (OXPHOS Complex V), as a cause of CMT2 further highlight the role of the mitochondria in the peripheral nerve axon.^21^ Similarly, the most common inherited optic neuropathies, Leber hereditary optic neuropathy (LHON) and autosomal dominant optic atrophy, are the result of mitochondrial dysfunction. LHON is caused by primary mitochondrial DNA (mtDNA) mutations affecting the respiratory chain complexes,^22^ while the majority of optic atrophy families have mutations in the *OPA1* gene, which encodes for an inner mitochondrial membrane protein important for mtDNA maintenance and oxidative phosphorylation.^23^ ^24^

Although we could not assess the activities of the mitochondrial respiratory chain complex c, II–III and IV, the decrease in mitochondrial respiration rate and the reduction in mitochondrial membrane potential point to a global and uniform mitochondrial respiration dysfunction, consistent with other reports describing loss of function mutations in *C12orf65* gene.^17^

CMT6 is a combination of two clinical processes caused by defective mitochondrial function that leads to axonal neuropathy and optic atrophy. The three genes identified in CMT6 confirm this and the phenotype is variable, a frequent finding in mitochondrial disorders. Overall CMT highlights the link between the axon and mitochondrial dysfunction and suggested that this process is likely to be affected in other forms of acquired axonal dysfunction such as multiple sclerosis and idiopathic neuropathy. From a genetic perspective, the combination of axonal neuropathy and CMT in any patient should alert the clinician to the possibility of a mitochondrial defect with a clear list of candidate genes to investigate.
